# Utility of Gynecological Teaching Associates

**DOI:** 10.7759/cureus.40601

**Published:** 2023-06-18

**Authors:** Katherine Kelly, Lauren Wilder, Jessica Bastin, Abbi Lane-Cordova, Bo Cai, James Cook

**Affiliations:** 1 Department of Obstetrics and Gynecology, Prisma Health Richland Hospital, Columbia, USA; 2 Department of Exercise Science, Arnold School of Public Health, University of South Carolina, Columbia, USA; 3 Department of Epidemiology and Biostatistics, Arnold School of Public Health, University of South Carolina, Columbia, USA; 4 Department of Medical Education and Academic Affairs, School of Medicine, University of South Carolina, Columbia, USA

**Keywords:** gta, medical school education, clinical skills, gynecological teaching associate, standardized patient, pelvic exam

## Abstract

Introduction

Gynecological teaching associates (GTAs) are trained to teach the pelvic exam using themselves as models, and it has been hypothesized that their use can improve learners’ confidence and interpersonal skills. This study aims to gain greater insight into whether the use of GTAs is associated with increased medical students’ confidence when performing the pelvic exam during clinical rotations.

Methods

An email survey was distributed to medical students in two different classes at a single United States Medical Licensing Examination (USMLE)-accredited medical school: one that learned the pelvic exam using GTAs and one that did not. A Fisher's exact test was performed to determine associations between the use of GTAs and confidence in performing the pelvic exam, with a p-value of <0.01.

Results

Out of the 85 survey participants, 68 had performed a pelvic exam in the clinical setting and thus rated their confidence level. Of the 38 students who learned using a GTA, 66% (p<0.0024) reported a confidence level of four or five (out of five) compared to 50% of the 30 students who were not able to practice using a GTA. There was a statistically significant difference in the confidence levels of students who practiced on GTAs compared to those who did not.

Discussion

Our findings demonstrated that students who were able to learn the pelvic exam using GTAs reported higher confidence levels when subsequently performing a pelvic exam in a clinical setting.

Conclusion

Our findings support investment in GTA programming for teaching the pelvic exam in medical school curricula.

## Introduction

The pelvic exam is an important clinical assessment that is typically taught during the second and third years of medical school. The exam can be used to assess the cause of bleeding, vaginal discharge, or pain and as a screening tool for cervical cancer and sexually transmitted infections [1]. Traditionally, the pelvic exam is taught using standardized patients (SPs) or gynecological teaching associates (GTAs) and by simulators or plastic models [2]. SPs are trained individuals who help to simulate real-world healthcare situations to improve confidence and skills among learners [3]. GTAs are a type of SP who are trained and educated to teach breast, speculum, and bimanual vaginal examinations using themselves as a demonstration and practice model [4,5]. GTAs allow the learner to examine her and provide immediate instruction and feedback regarding examination technique and communication skills [6].

Historically, medical students have found the pelvic exam to be intimidating and challenging due to the inherently sensitive nature of the exam [7]. Various training methods are used to allow students to learn in a comfortable and encouraging environment before they are expected to perform the exam on patients in a clinical setting. A best practice for teaching the pelvic exam has not yet been established, but previous studies have demonstrated that student use of SPs and GTAs led to better communication with patients and interpersonal skills compared to peers who trained using more traditional methods, such as with plastic models or clinic patients [8-10]. Although multiple studies have suggested the benefits of using SPs, a possible limitation of widespread use in medical curricula is that the cost of using SPs for student learning is much higher than other, more traditional teaching methods [11].

Many in-person activities, including those involving medical education, were paused during the 2020-2021 school year due to the COVID-19 pandemic restrictions. Prior to the pandemic, the pelvic exam had been taught at the University of South Carolina School of Medicine (USCSOM), Columbia, during the second year of medical school using a series of lectures followed by practice with GTAs. Due to the pandemic, the class of 2022 (fourth-year medical students) adopted a modified curriculum (MC). The MC group was still provided with the same didactic lectures and educational materials, but they were not able to experience any hands-on portions of the curriculum, such as GTAs. Conversely, the class of 2023 (third-year medical students) was provided with the standard curriculum (SC), which includes the use of GTAs, as the restrictions had lessened during this phase of their training. To gain insight into the impact of learning the pelvic exam on a GTA, we took advantage of these natural experiment conditions created by the pandemic and surveyed the class of 2022 and 2023 regarding their feelings of confidence and comfortability. We hypothesized that the students in the SC class would report greater confidence and comfortability than the MC class when subsequently performing the exam on patients in the clinical setting.

This article was previously presented as a poster presentation at the Director of Clinical Skills Annual Meeting on November 11, 2022, and as a poster presentation at Discover USC on April 21, 2023.

## Materials and methods

Study overview

An online survey was created using Google Forms and distributed via email to 96 third-year medical students (SC group) and 94 fourth-year medical students (MC group) at USCSOM, Columbia, in February of 2022. The email included information on the study design and purpose, the consent form, and the link to complete the Google Form. Data from the survey were exported to an Excel document. This study was approved by the University of South Carolina Institutional Review Board (Pro00117289).

Participants

Survey participants included third- and fourth-year English-speaking medical students attending the USCSOM, Columbia, who have completed their second year of medical school and have started clinical rotations. Those excluded from the study were those who did not have a device with the capability to complete an online survey, non-English-speaking students, and students under the age of 18.

Modified curriculum

An obstetrics and gynecology physician gave a one-hour lecture on performing the pelvic exam to students. It included female anatomy, tools used for the exam, method of performing the exam, and what to look for during the exam. Students watched the presentation virtually, and it was recorded for repeated viewing.

Standard curriculum

SC students were given the same pelvic exam presentation as the MC group. They could choose to watch the presentation in person or virtually, and it was recorded. A GTA program was hired by the medical school, and video demonstrations were provided to students by the program. GTAs taught groups of two students how to perform a thorough pelvic and breast exam using clinic exam rooms. Students practiced the exam on the GTAs, and the GTAs provided feedback on students’ technique and accuracy throughout the exam.

Survey

The survey contained four sections. The first section included questions about the participants’ demographic information, including age, race, gender, and year in medical school. All demographic questions were optional, and participants were given a "Prefer not to answer" option. The second section focused on the experiences of participants. Respondents reported whether they had the opportunity to practice the pelvic exam on a GTA, how many times they practiced, and what additional educational materials they used before performing the pelvic exam on a real patient. Respondents self-reported their opinion on how helpful the GTA experience was when learning the pelvic exam, which was measured on a Likert scale of one to five, with one being “strongly disagree” and five being “strongly agree.” The effect of the GTA experience on respondents’ comfort level when performing the pelvic exam was measured on a scale of one to five, with one being “did not increase comfort level at all” and five being “greatly increased comfort level.” The third section focused on clinical experience. Respondents reported if they had performed the pelvic exam on a patient in a clinical setting and self-reported their confidence in performing the exam. Confidence was measured on a scale of one to five, with one being “not confident” and five being “very confident.” In the fourth section of the survey, respondents accepted the terms and conditions that were supplied in the email they received and offered consent to be enrolled in the study. The survey did not obtain any identifying information.

Statistical analyses

A Fisher’s exact test was performed using SAS 9.4 statistical software to determine the association between students having the opportunity to practice the pelvic exam on a GTA and their confidence in performing a pelvic exam on patients during clinical rotations. The significance level was set at a p-value <0.01.

## Results

Participant characteristics

Out of the 190 students who were invited to participate in the survey, 85 chose to participate. Of these, approximately half were female (50.6%, n=43, Table 1) and half were male (45.9%, n=39); three eligible participants declined to answer (3.5%, n=3). The majority of respondents were between ages 24-26 (58.8%, n=50), followed by ages 27-29 (32.9%, n=28), and less than 5% reported their age as 21-23 (2.4%, n=2), 30-32 (3.5%, n=3), or 32+ (2.4%, n=2). Just over three-fourths of respondents were White (78.8%, n=67) and 13% were Asian (12.9%, n=11). Forty-eight (56.5%, n=48) respondents were in their third year of medical school (SC group), and 37 (43.5%, n=37) were in their fourth year (MC group).

**Table 1:** Respondents’ demographics. MIII: third-year medical student; MIV: fourth-year medical student.

	Frequency (n)	Percentage of respondents
Gender		
Female	43	50.59
Male	39	45.88
Prefer Not to Say	3	3.53
Total	85	100
Age (years)		
21-23	2	2.35
24-26	50	58.82
27-29	28	32.94
30-32	3	3.53
32+	2	2.35
Total	85	100
Race		
Asian	11	12.94
Black or African American	1	1.18
Other	3	3.53
Prefer not to say	3	3.53
White	67	78.82
Total	85	100
Class		
MIII	48	56.47
MIV	37	43.53
Total	85	100

Survey response analysis

When learning the pelvic exam, participants in both the MC and SC groups reported using lecture materials (72.9%, n=62, Table 2), outside or third-party resources (34.1%, n=29), anatomical models (17.6%, n=15), other resources (16.5%, n=14), or no other resources outside of a GTA (2.4%, n=2).

**Table 2:** Educational resources used to learn the gynecological exam, total number of respondents n=85.

Other educational resources used	Frequency (n)	Percentage of respondents
Lecture material	62	72.9
Outside resources	29	34.1
Anatomical model	15	17.6
Other	14	16.4
None	2	0.24

Greater than half of the participants (57.7%, n=49, Table 3) responded yes to learning the pelvic exam on a GTA and the remaining participants responded no (42.3%, n=36). The majority of respondents have performed a pelvic exam on a patient in a clinical setting (80.00%, n=68).

**Table 3:** Respondents’ self-reported experiences. SP: standardized patient.

	Frequency (n)	Percentage of respondents
Did you learn and practice the pelvic exam on a SP?		
No	36	42.35
Yes	49	57.65
Total	85	100
Have you performed a pelvic exam on a patient in a clinical setting?		
No	17	20
Yes	68	80
Total	85	100

**Figure 1:**
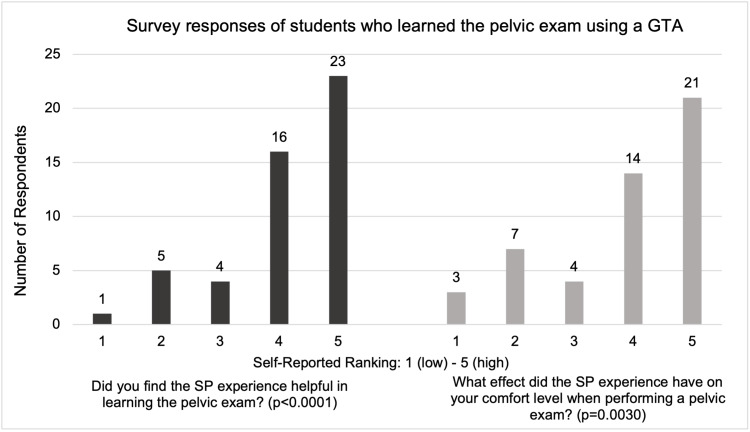
Survey responses of students who learned the pelvic exam using a GTA. GTA: gynecological teaching associate.

Among the 49 respondents who learned the pelvic exam using a GTA, 39 participants (79.6%, n=39, p<0.0001) rated the helpfulness of the GTA experience as a four or above, and 10 participants (20.4%, n=10) rated the helpfulness as a three or below (Figure 1). Thirty-five participants (71.4%, n=35, p=0.0030) who had the GTA experience described their comfort level when performing the pelvic exam on a patient in a clinical setting as a four or above, while only 14 (28.6%, n=14) responded with a three or below.

Bivariate analysis

**Figure 2:**
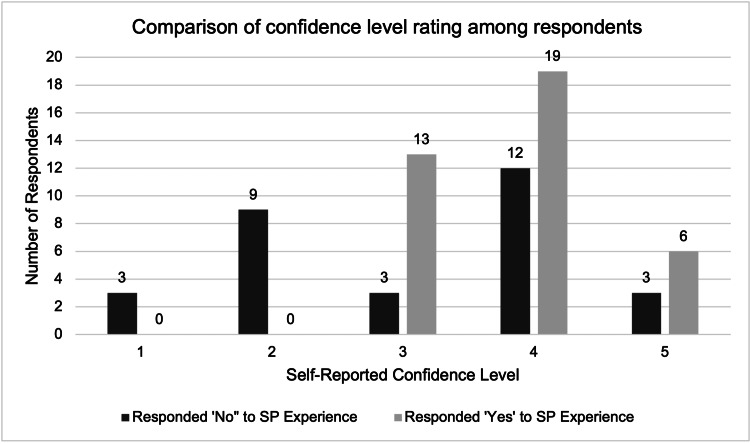
Comparison of confidence level ratings among participants who responded "no" to the SP experience compared to those who responded "yes," p<0.0024. SP: standardized patient.

Out of the 85 survey participants, 68 reported that they had performed a pelvic exam on a patient in a clinical setting and thus rated their confidence level regarding performing the exam. Out of 38 students in the SC group, nearly two-thirds (65.8%, n=25, p<0.0024; Figure 2) reported a confidence level of four or five (out of five), while only half (50.0%, n=15, p<0.0024) of the 30 students in the MC group reported a confidence level of four or five (out of five). These figures were calculated using the survey answers of everyone who rated their confidence in performing a pelvic exam in the clinical setting. There was a significant difference in the odds of reporting a confidence level of four or five (out of five) in those in the SC group compared to the MC group (OR=1.85, 95% CI [0.72, 4.77]). There was no significant association found for age, gender, or race with the student’s level of comfort when performing a pelvic exam. Additionally, no significant association was found when comparing age, gender, or race with study participants' confidence levels. 

## Discussion

Results from this study supported our hypothesis and demonstrated that students who were able to learn and practice the pelvic exam on a GTA reported higher confidence levels than those who did not when subsequently performing a pelvic exam in a clinical setting. Additionally, the majority of students reported that the GTA experience was helpful when learning the technique of the pelvic exam.

This study expanded upon prior research that investigated the benefits and disadvantages of various teaching methods when learning the pelvic exam during medical school. Previous studies have suggested that using GTAs while learning the pelvic exam led to less anxiety and greater confidence among students [7,12]. Additionally, students who were instructed by a GTA demonstrated better communication skills with patients [7]. It has also been shown that students who learned using a GTA performed more pelvic exams during their clinical clerkship compared to students who did not [13]. Maximizing patient comfort during a sensitive exam such as the pelvic exam is important, especially for adolescent and menopausal women, sexual minorities, obese women, women with disabilities, and women with a history of trauma or prior instrumentation affecting the genitalia [1]. Students who display confidence and communicate effectively can better foster a comfortable environment for an exam that is usually perceived as uncomfortable by patients.

One study found that students who participated in a pelvic examination teaching program using well-women from the general community also reported improved confidence and reduced anxiety [14]. This study could suggest that increased opportunities to perform pelvic examinations account for decreased student anxiety and greater confidence, regardless of whether the exam is done using a GTA or untrained women from the community. Another study found that while practicing a pelvic exam on a GTA was associated with increased competence and communication skills, there was no effect on student confidence overall [15]. This may also indicate that the number of opportunities to practice pelvic exams has the most influential impact on student confidence rather than the GTA experience itself. Additionally, it must be considered that self-reported confidence levels have not been proven to positively correlate with improved technical skills [16]. However, medical students with greater confidence have been shown to perform a greater number of examinations during their clinical clerkships [14]. This repetition of exam skills can reinforce techniques to increase the probability of detecting pathology when present.

Typically, depriving a cohort of students of the opportunity to learn the pelvic exam using a GTA would be inequitable and unethical. This type of study was only possible due to the unique situation created by the unexpected consequences of the COVID-19 pandemic. While a gold standard has yet to be identified, additional research would be beneficial to identify a comparable alternative teaching method as a low-cost alternative or in the event of another unprecedented event. There are studies investigating the growing role of simulation in obstetrics and gynecological training. An expert review article published by the American Journal of Obstetrics and Gynecology states that using a hybrid model with an inanimate pelvic model for examination and a GTA for the history-taking portion of the pelvic exam provides an acceptable and cost-effective alternative to performing the full pelvic exam on a GTA [17]. Additionally, 3D virtual reality pelvic models and simulation models with newer, advanced technology are both viable options for teaching the pelvic exam to students without the use of a GTA [18].

Our study results should be interpreted in the context of its limitations: We only conducted the study at a single university located in the southeastern United States. Our student participants were primarily White or Asian and under 30 years old; results may not be generalizable to other geographic, race, or age groups. Additionally, the data for this study were based on self-reported analysis, which is a subjective measure whose interpretation can vary from person to person.

To our knowledge, this survey was the first of its kind to evaluate students' opinions of learning the pelvic exam who are participating in the same curriculum at a single medical school. We were also able to capture opinions from the unique subset of the student population whose education was impacted by curriculum and procedural changes in response to COVID-19. Our findings support the use of GTAs in teaching pelvic exams, as more comfortable and confident students promote better health outcomes for patients. Further research is necessary to determine the gold standard for learning and gaining confidence in performing the pelvic exam to modify and standardize curricula across medical education.

## Conclusions

The pelvic exam is an important service to provide for patients, but it can be a daunting skill to learn for medical students and an uncomfortable experience for patients. Our survey showed that students felt more comfortable and confident performing a pelvic exam on a patient in a clinical setting after they learned the technique from a GTA rather than through written or video resources alone. These findings support investment in GTA programming for teaching the pelvic exam in preclinical medical school curricula.
